# The development of the ICD-11 chapter on mental disorders

**DOI:** 10.4102/sajpsychiatry.v23.i0.1105

**Published:** 2017-05-09

**Authors:** Umberto Volpe

**Affiliations:** 1 Department of Psychiatry, University of Naples SUN, Italy

The 11th edition of the International Classification of Diseases and Related Health Problems (ICD-11) is expected to be approved by the World Health Assembly in May 2018.

The chapter on mental disorders will include the following groupings: neurodevelopmental disorders, schizophrenia and other primary psychotic disorders, mood disorders, anxiety and fear-related disorders, obsessive–compulsive and related disorders, disorders specifically associated with stress, dissociative disorders, bodily distress disorders, feeding and eating disorders, elimination disorders, disorders due to substance use, impulse control disorders, disruptive behaviour and dissocial disorders, personality disorders, paraphilic disorders, factitious disorders, neurocognitive disorders, and mental and behavioural disorders due to disorders or diseases classified elsewhere.

Sleep-wake disorders and conditions related to sexual health will be covered in separate chapters of the classification. This decision has been taken in order to overcome the ICD-10 distinction, regarded as obsolete, between ‘organic’ and ‘non-organic’ sleep disorders (included, respectively, in the chapters on diseases of the nervous system and on mental and behavioural disorders) and between ‘organic’ and ‘non-organic’ sexual dysfunctions (included, respectively, in the chapters on diseases of the genito-urinary system and on mental and behavioural disorders).

The proposed ICD-11 diagnostic guidelines subdivide sexual dysfunctions into four main groups: sexual desire and arousal dysfunctions, orgasmic dysfunctions, ejaculatory dysfunctions and other specified sexual dysfunctions. The conditions that appeared as gender identity disorders in ICD-10 have been reconceptualised as ‘gender incongruence’ and also proposed to be moved to the new chapter on sexual health. The ICD-10 categories related to sexual orientation have been recommended for deletion from the ICD-11.^1,2,3^

For each disorder included in the above-mentioned groupings, the ICD-11 clinical descriptions and diagnostic guidelines will provide (1) a brief definition (100–125 words); (2) a list of inclusion and exclusion terms; (3) a description of the essential (required) features, that is, those characteristics that a clinician could expect to find in all cases of the disorder; (4) a guidance concerning the differentiation between the disorder and some relevant ‘normal’ conditions (‘boundary with normality’); (5) a list of the disorders that should be distinguished from the one being described and a guidance on how to make the differential diagnosis (‘boundary with other disorders’); (6) coded qualifiers and subtypes; (7) clinically relevant information regarding the typical course (‘course features’); (8) ‘associated clinical presentations’ (i.e. clinically important conditions that are frequently associated with the disorder, which may require their own assessment and treatment); (9) culture-related features; (10) developmental presentations (i.e. a description of how the disorder may present differently according to the developmental stage of the individual, including childhood, adolescence and older adulthood); and (11) gender-related features.^4^

A draft of the ICD-11 clinical descriptions and diagnostic guidelines has been developed by the relevant working groups for all the disorders included in the above-mentioned groupings.^5^ A simplified draft of the guidelines has been produced for use in ICD-11 field studies.^6^ This simplified draft contains, for each disorder, a brief definition, a description of the essential (required) features, a section on the boundary with other disorders and with normality and a description of the most commonly associated features. For some of the disorders, qualifiers or subtypes are also provided. At the moment, this simplified version of the clinical descriptions and diagnostic guidelines is available for schizophrenia and other primary psychotic disorders, mood disorders, anxiety and fear-related disorders, disorders specifically associated with stress, and feeding and eating disorders. For all other groupings, a brief general definition and sometimes a description of some of the disorders included in the grouping can be found on the ICD-11 beta platform.^7^

All these materials are not to be regarded as definitive. Indeed, the World Health Organization (WHO) welcomes comments and suggestions from the field.^4,6^ In order to collect these comments and suggestions, the WHO has created an Internet platform called GCP. This platform provides a network that can be accessed by all the members of the Global Clinical Practice Network. This network can be joined by all mental health or primary care professionals who are legally authorised to provide services to people with mental and behavioural disorders in their countries. At present, the network consists of more than 12 500 mental health and primary care professionals from almost 150 countries, over half of whom are psychiatrists^6^ (see http://gcp.network to register in any of the nine languages).

Internet-based and clinic-based field studies aiming to test the ICD-11 draft of the clinical descriptions and diagnostic guidelines for the various disorders are now ongoing.^8,9^ Internet-based field studies use a case vignette methodology to examine clinical decision-making in relation to the proposed ICD-11 guidelines and are being conducted through the Global Clinical Practice Network. Clinic-based field trials aim to assess the reliability and utility of the proposed ICD-11 guidelines in the clinical settings where the classification will be used and are being conducted through the WHO Network of International Field Study Centres.

Although clinical utility has been emphasised as a primary objective of the previous versions of the ICD as well as of the DSM-III and its successors,^4,10,11,12^ and has been often regarded as the highest priority in diagnostic systems,^13^ this is in fact the first time that the clinical utility of a psychiatric diagnostic system is being tested systematically.

Throughout the ICD-11 draft, the description of the essential (required) features of various mental disorders usually lack the specific thresholds concerning number and duration of symptoms that characterise the DSM-III and its successors. Instead, the diagnostic guidelines are intended to conform to the way psychiatrists actually make diagnoses in ordinary practice, that is, by the flexible exercise of clinical judgement.^4^

The possibility of a dialogue between the ICD revision process and the Research Domain Criteria (RDoC) project launched by the US National Institute of Mental Health is being considered. Indeed, the main objectives of the two projects (i.e. improving the clinical utility of psychiatric diagnoses for the ICD and exploring in an innovative way the etiopathogenetic bases of psychopathology for the RDoC) can be regarded as complementary, and much can be done to reduce the current gap between the RDoC constructs and the clinical phenomena that psychiatrists encounter in their ordinary practice, especially in the area of psychoses.^14,15^

## References

[1] Cochran SD, Drescher J, Kismödi E, et al. Proposed declassification of disease categories related to sexual orientation in the International Statistical Classification of Diseases and Related Health Problems (ICD-11). Bull World Health Organ. 2014;92:672–679. 10.2471/BLT.14.135541 25378758 PMC4208576

[2] Chou D, Cottler S, Khosla R, Reed GM, Say L Sexual health in the International Classification of Diseases (ICD): Implications for measurement and beyond. Reprod Health Matters. 2015;23:185–192. 10.1016/j.rhm.2015.11.008 26719010

[3] Reed GM, Drescher J, Krueger RB, et al. Disorders related to sexuality and gender identity in the ICD-11: Revising the ICD-10 classification based on current scientific evidence, best clinical practices, and human rights considerations. World Psychiatry. 2016;15:205–221. 10.1002/wps.20354 27717275 PMC5032510

[4] First MB, Reed GM, Hyman SE, Saxena S The development of the ICD-11 Clinical Descriptions and Diagnostic Guidelines for Mental and Behavioural Disorders. World Psychiatry. 2015;14:82–90. 10.1002/wps.20189 25655162 PMC4329901

[5] Reed GM Toward ICD-11: Improving the clinical utility of WHO’s International Classification of Mental Disorders. Prof Psychol Res Pract. 2010;41:457–464. 10.1037/a0021701

[6] Reed GM, First MB, Medina-Mora ME, Gureje O, Pike KM, Saxena S Draft diagnostic guidelines for ICD-11 mental and behavioural disorders available for review and comment. World Psychiatry. 2016;15:112–113. 10.1002/wps.20322 27265692 PMC4911773

[7] Luciano M The ICD-11 beta draft is available online. World Psychiatry. 2015;14:375–376. 10.1002/wps.20262 26407802 PMC4592669

[8] Evans SC, Roberts MC, Keeley JW, et al. Vignette methodologies for studying clinicians’s decision-making: Validity, utility, and application in ICD-11 field studies. Int J Clin Health Psychol. 2015;15:160–170. 10.1016/j.ijchp.2014.12.001 PMC6224682 30487833

[9] Keeley JW, Reed GM, Roberts MC, et al. Developing a science of clinical utility in diagnostic classification systems: Field study strategies for ICD-11 mental and behavioural disorders. Am Psychol. 2016;71:3–16. 10.1037/a0039972 26766762

[10] First MB Clinical utility in the revision of the Diagnostic Statistical Manual of Mental Disorders (DSM). Prof Psychol Res Pract. 2010;41:465–473. 10.1037/a0021511

[11] First MB, Bhat V, Adler D, et al. How do clinicians actually use the Diagnostic and Statistical Manual of Mental Disorders in clinical practice and why we need to know more. J Nerv Ment Dis. 2014;202:841–844. 10.1097/NMD.0000000000000210 25390931

[12] Roberts MC, Reed GM, Medina-Mora ME, et al. A global clinicians’ map of mental disorders to improve ICD-11: Analysing meta-structure to enhance clinical utility. Int Rev Psychiatry. 2012;24:578–590. 10.3109/09540261.2012.736368 23244613

[13] Jablensky A Psychiatric classifications: Validity and utility. World Psychiatry. 2016;15:26–31. 10.1002/wps.20284 26833601 PMC4780305

[14] Maj M Narrowing the gap between ICD/DSM and RDoC constructs: Possible steps and caveats. World Psychiatry. 2016;15:193–194. 10.1002/wps.20370 27717257 PMC5032499

[15] Sanislow CA Updating the research domain criteria. World Psychiatry. 2016;15:222–223. 10.1002/wps.20374 27717261 PMC5032502

